# Homozygous *IL37* mutation associated with infantile inflammatory bowel disease

**DOI:** 10.1073/pnas.2009217118

**Published:** 2021-03-04

**Authors:** Zinan Z. Zhang, Yu Zhang, Tingyan He, Colin L. Sweeney, Safa Baris, Elif Karakoc-Aydiner, Yikun Yao, Deniz Ertem, Helen F. Matthews, Claudia Gonzaga-Jauregui, Harry L. Malech, Helen C. Su, Ahmet Ozen, Kenneth G. C. Smith, Michael J. Lenardo

**Affiliations:** ^a^National Institute of Allergy and Infectious Diseases, NIH, Bethesda, MD 20892;; ^b^Cambridge Institute of Therapeutic Immunology and Infectious Disease, University of Cambridge, Cambridge CB2 0AW, United Kingdom;; ^c^School of Medicine, Marmara University, 34722 Istanbul, Turkey;; ^d^Regeneron Genetics Center, Tarrytown, NY 10591

**Keywords:** immunodeficiency, inflammatory bowel disease, IBD, IL37, VEO-IBD

## Abstract

Interleukin (IL)-37, an antiinflammatory IL-1 family cytokine, is a key suppressor of innate immunity. IL-37 signaling requires the heterodimeric IL-18R1 and IL-1R8 receptor, which is abundantly expressed in the gastrointestinal tract. Here we report a 4-mo-old male from a consanguineous family with a homozygous loss-of-function *IL37* mutation. The patient presented with persistent diarrhea and was found to have infantile inflammatory bowel disease (I-IBD). Patient cells showed increased intracellular IL-37 expression and increased proinflammatory cytokine production. In cell lines, mutant IL-37 was not stably expressed or properly secreted and was thus unable to functionally suppress proinflammatory cytokine expression. Furthermore, induced pluripotent stem cell–derived macrophages from the patient revealed an activated macrophage phenotype, which is more prone to lipopolysaccharide and IL-1β stimulation, resulting in hyperinflammatory tumor necrosis factor production. Insights from this patient will not only shed light on monogenic contributions of I-IBD but may also reveal the significance of the IL-18 and IL-37 axis in colonic homeostasis.

Infantile inflammatory bowel disease (I-IBD) is defined by the onset of Crohn’s disease, ulcerative colitis disease, or IBD-unclassified disease in children under the age of 2 years old. I-IBD often presents with severe colitis that is refractory to treatments, immunodysregulation, and is etiologically linked to Mendelian mutations (1). Monogenic causes of very-early-onset IBD (VEO-IBD) include interleukin (IL)-10 and IL-10R deficiencies (1, 2). IL-10 is critical for dampening inflammatory responses in the gastrointestinal (GI) tract by inhibiting tumor necrosis factor (TNF) and IL-12 signaling (2). Defects in this pathway can lead to unchecked gut inflammation.

IL-37 plays an antiinflammatory role in the innate immune response. It is an IL-1 family cytokine that suppresses inflammation, unlike the other proinflammatory members in this gene family. IL-37 is predominantly expressed by macrophages or dendritic cells and can suppress TNF, IL-1β, and IL-6 signaling (3). The IL-37 receptor consists of IL-1R8 and IL-18R1, which are highly expressed in the GI tract (4). Interestingly, IL-37 signaling can occur via two mechanisms: intracellularly through nuclear translocation with SMAD3 and extracellularly after secretion and binding to its receptor (4). While mice do not normally express IL-37, transgenic mice with overexpressed human IL-37 are protected from lipopolysaccharide (LPS)-induced shock and dextran sulfate sodium–induced colitis (3, 5). In addition, IL-1R8 and IL-18R1/IL-18BP-knockout mice studies demonstrate how perturbation of the IL-1 and IL-18 axis can lead to increased inflammation and colonic disease (6, 7). Preliminary studies in humans show that heterozygous *IL37* gene variants may be associated with joint inflammation (8), and expression levels may be correlated with IBD outcome (9). Common *IL37* variants also modulate the activity of the cytokine (10). To date, however, no monogenic link has been shown between *IL37* and I-IBD, and the physiological function of IL-37 in the human body has not been established. Here, we report the case of a homozygous loss-of-function *IL37* variant associated with I-IBD.

Patient A1 is a 2-year-old boy born to a consanguineous Turkish family who presented at 4 mo of age with recurrent bloody diarrhea eight to nine times per day. His weight and length were in the 10th percentile. Laboratory findings (Dataset S1) were notable for anemia, leukocytosis, and elevated erythrocyte sedimentation rate and C-reactive protein. All microbiology tests were negative. His immunophenotyping profile was within normal limits for his age: normal proportions of monocytes, CD4^+^, and CD8^+^ T cells and a large proportion of undifferentiated naïve T and B cells. While his peripheral blood phenotyping appeared normal at 17 mo of age, abnormalities may manifest as A1 ages. A1 was initially maintained on a hypoallergenic diet due to concerns for food allergies, but colonoscopy findings of diffuse ulcers with wide-based crater formation throughout the colon and rectum, yet a normal appearing ileum, supported the diagnosis of infantile ulcerative colitis. Furthermore, histopathology of colon biopsies (Fig. 1*A*) showed diffuse and extensive lymphoplasmocytic infiltration, cryptitis, and apoptotic crypt abscesses throughout the colon and rectum, confirming the diagnosis of I-IBD. A1 was then treated with mesalamine and corticosteroids. After resolution of his GI symptoms and marked improvement observed by colonoscopy and histopathology (Fig. 1*A*), the patient was placed on a 5-mo steroid taper and azathioprine. A1 also had mild motor developmental delay but no additional medical problems.

**Fig. 1.**
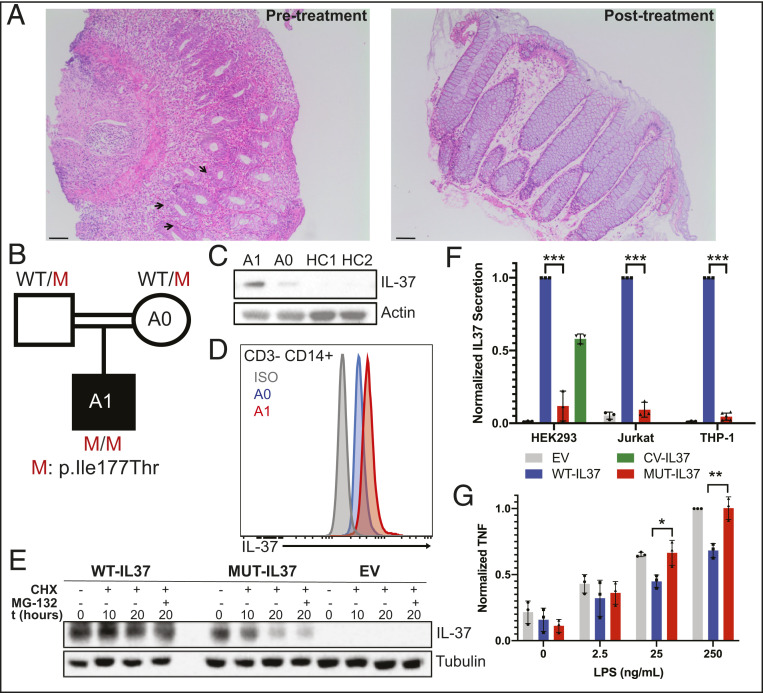
(*A*) Photomicograph (10×) of hematoxylin and eosin–stained colon sections of patient A1 pretreatment and posttreatment; arrows indicate cryptitis. (Scale bars, 100 μm.) (*B*) Pedigree of patient with *IL37* mutation. M = mutant. (*C*) Immunoblot of PBMCs from patient A1, control mother A0, and two unrelated healthy controls (HC). (*D*) Flow cytometry histogram of intracellular IL-37 expression in CD3^−^ CD14^+^ cells; red, homozygous affected A1; blue, heterozygous healthy mother A0; gray, isotype control. (*E*) Immunoblot of cycloheximide chase assay of HEK293T cells transfected with pLTV-WT-IL37, pLTV-MUT-IL37, or empty vector (EV); CHX = cycloheximide; MG-132 = proteasome inhibitor; t = time. (*F*) Graph of secreted supernatant IL-37 levels in transfected HEK293T cells, transduced Jurkat cells, and transduced THP-1 cells; gray, empty vector (EV); blue, pLTV-WT-IL37; red, pLTV-MUT-IL37; green, CV-IL37. (*G*) Graph of secreted TNF of LPS-stimulated (4 h), post-PMA differentiated (24 h), transduced THP-1 cells; black, empty vector (EV); blue, pLTV-WT-IL37; red, pLTV-MUT-IL37. *P < 0.05; **P < 0.01; ***P > 0.001. Mann–Whitney *U* tests were performed in GraphPad Prism. Experiments were performed at least three times in triplicate.

Given the familial consanguinity and diagnosis of I-IBD, we performed trio-based whole-exome sequencing (WES) analysis on the kindred (11) to search for a monogenic cause of disease. We found no rare variants in known VEO-IBD disease-causing genes. However, the *IL37* chr2: g.113676259 T > C (c.530T > C; p.Ile177Thr) missense variant (Fig. 1*B*) was prioritized based on a homozygous recessive model of inheritance analysis. Other identified rare homozygous variants are listed in Dataset S2. The *IL37* variant has a minor allele frequency of 0.000007073 with no reported homozygotes in the Genome Aggregation Database and a Combined Annotation Dependent Depletion score of 23, which is above the 99% confidence interval of the mutation significance cutoff of 3.313. The patient’s homozygous *IL37* variant was confirmed by Sanger sequencing analysis, and the mother (A0) was confirmed to be heterozygous for the variant (*SI Appendix*, Fig. S1*A*). Crystal structure modeling shows that the variant introduces a polar threonine residue in the place of a nonpolar isoleucine in the β-barrel domain of IL-37, which is likely to destabilize the protein structure to promote solvent accessibility of the substituted polar residue (*SI Appendix*, Fig. S1*B*).

Based on these genetic results, we evaluated IL-37 protein expression and stability. Interestingly, A1’s peripheral blood mononuclear cells (PBMCs) showed higher levels of IL-37 protein by immunoblot compared to healthy controls or A0 (Fig. 1*C*). Moreover, flow cytometric analysis showed patient monocytes had higher levels of intracellular IL-37 compared to A0 (Fig. 1*D*). Despite the increased protein expression of I177T IL-37, cycloheximide chase assay results indicated that the mutant Ile177Thr IL-37 (MUT-IL37) was less stable than wild-type IL-37 (WT-IL37) (Fig. 1*E*). In particular, after cells were incubated for 20 h with a translation inhibitor (cycloheximide) and a proteasome inhibitor (MG-132), mutant IL-37 was readily eliminated via endoplasmic reticulum–associated degradation. Although there may be a compensatory mechanism to increase mutant IL-37 expression, the protein is less stable and targeted for degradation.

To further examine the function of MUT-IL37, we assessed its translocation and potential antiinflammatory role. At basal level, primary monocyte-derived dendritic cells (moDCs) from A1 appeared to produce more TNF and interferon gamma (IFNγ) relative to healthy controls (*SI Appendix*, Fig. S1*C*). In HEK293T, Jurkat, and THP-1 cells—transfected or transduced with either WT-IL37 or MUT-IL37—there were consistently very low levels of MUT-IL37 secretion across all cell lines relative to common variant (CV) IL37 and WT-IL37 secretion (Fig. 1*F*). Moreover, caspase-1 processed MUT-IL37 has difficulty translocating to the nucleus after IL-1β stimulation relative to WT-IL37 (*SI Appendix*, Fig. S1*D*). Since MUT-IL37 cannot be stably expressed or properly translocated, it also cannot function properly to inhibit proinflammatory cytokines. While phorbol 12-myristate 13-acetate (PMA)-differentiated THP-1 cells transduced with WT-IL37 were capable of suppressing TNF levels in response to LPS stimulation, cells transduced with MUT-IL37 were unable to do so (Fig. 1*G*). Thus, the I177T mutation confers loss of functional IL-37 activity.

Due to limited availability of patient innate immune cells, we reprogrammed A1 and familial healthy control A0 PBMCs into induced pluripotent stem cells (iPSCs) and differentiated the iPSCs into macrophages. A1 iPSC-derived macrophages are more readily activated by LPS stimulation and have increased CD80 and CD38 expression at baseline and with stimulation relative to A0 (Fig. 2*A*). Interestingly, the iPSC macrophages from A1 have reduced major histocompatibility complex class II HLA-DR expression both before and after stimulation relative to the control (Fig. 2*B*). Intracellular staining of iPSC macrophages corroborates the increased IL-37 intracellular expression in the A1 primary monocytes (Fig. 2*C*). Moreover, A1 has increased expression of IL-1R3 subunit of the IL-1 receptor and IL-18R1 subunit of the IL-37 receptor, while expression of the other component of the IL-37 receptor, IL-1R8, is comparable to the control (Fig. 2*C*). Functionally, the patient’s iPSC-derived macrophages significantly overproduce TNF in response to both LPS and IL-1β stimulation (Fig. 2*D*). This highlights the role of IL-37 in suppressing inflammation and that the *IL37* mutation causes activated, hyperinflammatory macrophages.

**Fig. 2.**
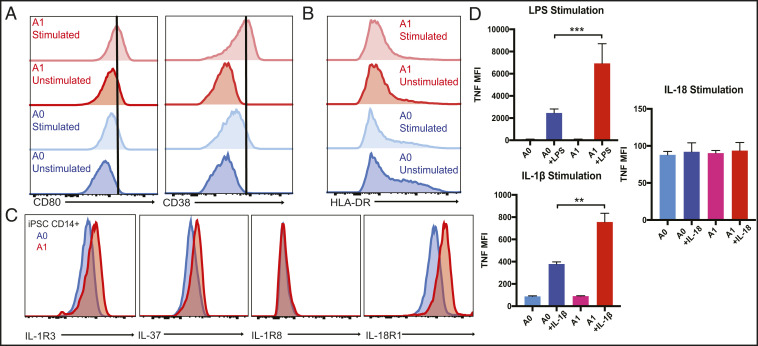
(*A*) Histograms of CD80 and CD38 expression of LPS- (100 ng/mL) stimulated (4 h) iPSC-derived macrophages from the patient A1 and heterozygous mother A0. (*B*) Histogram of HLA-DR expression of LPS- (100 ng/mL) stimulated (4 h) iPSC-derived macrophages. (*C*) Histograms of intracellular expression of IL37 and surface expression of IL-1R3, IL-1R8, and IL-18R1 of A1 and A0 iPSC-derived macrophages. (*D*) Graphs of TNF production by iPSC macrophages stimulated with LPS (100 ng/mL), IL-1b (10 ng/mL), and IL-18 (10 ng/mL). Mann–Whitney *U* test, **P < 0.01, ***P < 0.001.

In conclusion, we establish that a physiological role of IL-37 in the human body is to establish immunological tolerance in the GI tract by this report of autosomal recessive IL-37 deficiency leading to I-IBD. While protein encoding I177T IL-37 is expressed, it is not stable, cannot be secreted, and appears unable to suppress proinflammatory signals. Unlike VEO-IBD patients with IL10/IL10R mutations, who are often difficult to treat and may need hematopoietic stem cell transplants, the disease in this patient has been well controlled for over a year now on standard immunosuppressive treatments with complete resolution of GI symptoms. Although it is premature to predict what the long-term outcome of IL-37 deficiency will be, it is interesting to note that the patient has had only GI symptoms to date without any broader immunodeficiencies. Moreover, given the increased TNF levels, if the patient relapses, he may specifically benefit from TNF-blockade therapy. Nonetheless, genetic screening for *IL37* deleterious variants may be beneficial for at-risk families. The identification of additional families will help to define the natural history of this disorder. This research sheds light on the role of IL-37 in homeostatic control of colonic inflammation in humans and begs the question of whether IL-37 can be used as a novel cytokine therapeutic for IBD and potentially other inflammatory conditions.

## Methods

Written informed consent for human subjects or their legal guardians was obtained by an NIH Institutional Review Board–approved protocol. DNA from the family was analyzed by WES. *SI Appendix* has detailed methods for IL-37 gene analyses.

## Supplementary Material

Supplementary File

Supplementary File

Supplementary File

## Data Availability

Genomic data have been deposited in dbGaP (phs002040.v1.p1).
