# Supplementary material for: Homozygous IL37 mutation associated with infantile inflammatory bowel disease
Source: Proc Natl Acad Sci U S A. 2021 Mar 4;118(10):e2009217118. doi: 10.1073/pnas.2009217118 (PMC7958356; doi:10.1073/pnas.2009217118)
Supplement: Supplementary File [file pnas.2009217118.sapp.pdf]

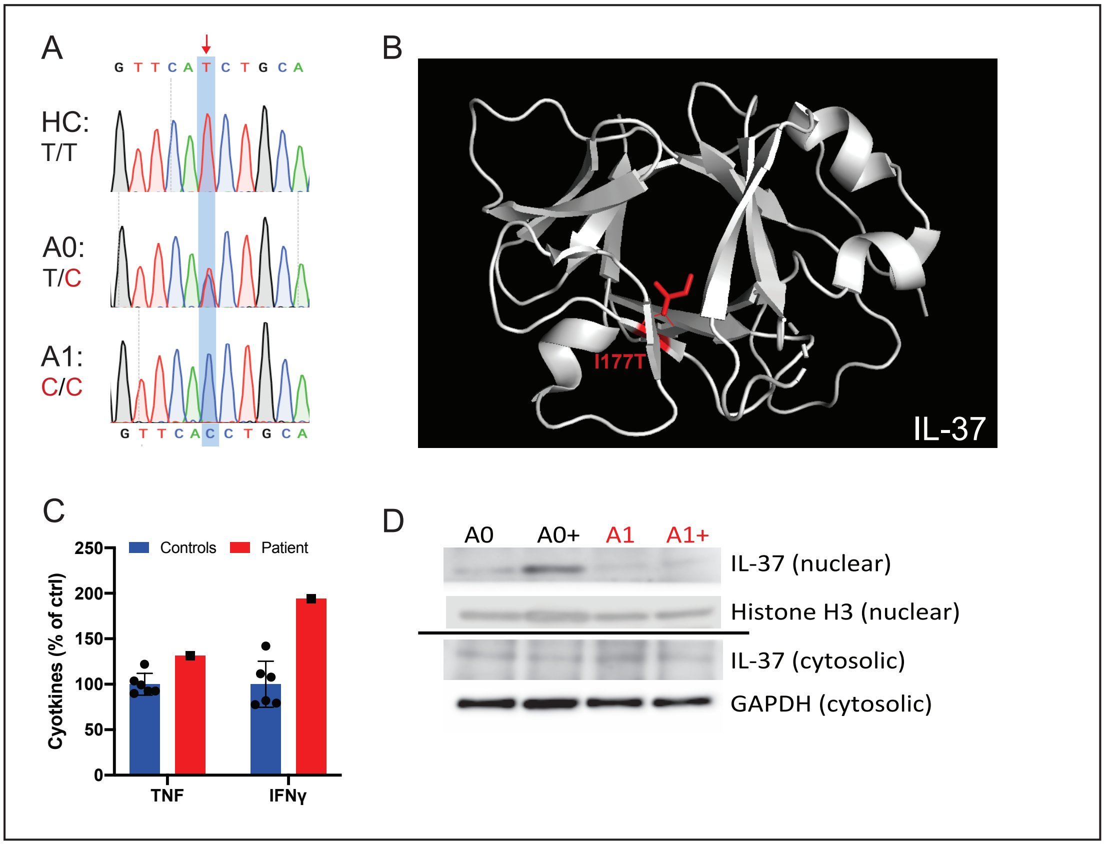

SI Appendix 1. (A) Sanger sequencing traces of healthy control, heterozygous mother A0, and homozygous patient A1. (B) Crystal structure of IL-37 (white) with the position of I177T mutation highlighted (red) (modified from Protein Data Bank ID code 5HN1; ref.11). (C) Graph of TNF and IFNγ secreted by moDCs from patient A1 and healthy controls. (D) Immunoblot of nuclear and cytosolic fraction of unstimulated and IL-1β-stimulated iPSC-derived macrophages from patient A0 and heterozygous control A1. + denotes stimulated with IL-1β (10 ng/mL for 24 hours). GAPDH, glyceraldehyde-3-phosphate dehydrogenase.

Methods

Human Subjects
Written informed consent was provided by all human subjects or their legal guardians in accordance with the 1975 Helsinki principles for enrollment in research protocols that were approved by the Institutional Review Board of the National Institute of Allergy and Infectious Diseases, National Institutes of Health (NIH). Patient and healthy control blood was obtained in Marmara University Faculty of Medicine in Istanbul, Turkey or at NIH under approved protocols. Members of the general public are involved in the institutional review board and therefore participate in the design of the study protocol used in this research.

Genetic Analysis
DNA was obtained from the proband and family members by isolation and purification from peripheral blood mononuclear cells (PBMCs) using Qiagen's DNeasy Blood and Tissue Kit. The DNA was then submitted for whole exome sequencing (WES) by Illumina sequencers in the United States. The reads were filtered for sequence quality and then mapped on to the h19 human genome reference by Burrows-Wheeler Aligner with default parameters. Alignment, variant calling, and annotation were performed by the in-house bioinformatics core using the Genome Analysis Toolkit version 3.4 (Broad Institute) and GEMINI (GEnome MINIng). The IL37 variant was confirmed by Sanger sequencing using following PCR primers:
F: ttatagggctcaggtgggct and R: acagactcagccacttctgc (Sigma Aldrich) or F: gtaccaaggctgacacgtca and R: agcagaagccggtctctttc (Sigma Aldrich).

Cells and Media
Primary patient or control PBMCs were obtained from whole blood subjected to Histopaque/Ficoll density gradient separation. The PBMCs were then washed with PBS and frozen in complete RPMI with 10% DMSO in liquid nitrogen for later use or -80°C for transport. HEK293T, Jurkat, and THP-1 cells were obtained from the American Type Culture Collection and tested mycoplasma-free (ATCC). Human cells were cultured in RPMI or DMEM (Sigma Aldrich) supplemented with 10% heat-inactivated fetal bovine serum (Sigma Aldrich), 1% penicillin/streptomycin (Gibco), and 1% Glutamax (Gibco). Primary human monocytes were isolated using magnetic isolation kit (Miltenyi) and cultured in complete RPMI supplemented with IL-4 (20 ng/mL, PeproTech) and GM-CSF (20 ng/mL, PeproTech). iPSCs and iPSC-derived macrophages were cultured in specialized media described below.

Antibodies
The following primary anti-human antibodies were used for Western blot analysis: polyclonal goat anti-IL37 (R&D Systems), polyclonal rabbit anti-IL37 (Invitrogen), polyclonal rabbit anti-beta actin (Abcam), and monoclonal mouse anti-beta tubulin (Biolegend). Secondary HRP-linked anti-rabbit IgG, anti-goat IgG, and anti-mouse IgG antibodies (CST) were used to conjugate to the respective primary antibodies. The following antibodies were used for flow cytometry analysis: IL-37 PE (37D12) (Invitrogen), CD3 PerCp-Cy5.5 (UCHT1) (Biolegend), and CD14 FITC (63D3) (Biolegend), CD80 FITC (Biolegend), CD38 PE (Biolegend), HLA-DR BV785 (Biolegend), IL-1R3 PE (R&D Systems), IL-1R8 APC (R&D Systems), and IL-18R1 FITC (R&D Systems). Cell viability was assessed using LIVE/DEAD Fixable Aqua (Invitrogen).

Reprogramming of Induced Pluripotent Stem Cells (iPSCs)
Transgene-free iPSCs were generated from peripheral blood CD34+ hematopoietic stem/progenitor cells (HSPCs) as previously described (Merling et al. *Blood* 2013), from either healthy volunteers or the IL37-deficient patient. Briefly, CD34+ HSPCs were purified from peripheral blood mononuclear cells using magnetic-activated cell sorting with CD34 microbeads (Miltenyi), followed by transduction with non-integrating CytoTune-iPS 2.0 Sendai viruses (Invitrogen), and then cultured in StemFit Basic02 feeder-free culture medium (amsbio) supplemented with 10 ng/mL human basic fibroblast growth factor (PeproTech) on plates coated with ESC-qualified Matrigel (Corning) for formation of iPSC colonies during initial reprogramming. For routine maintenance, iPSCs were cultured on Matrigel-coated plates in mTeSR1 or mTeSR Plus medium (STEMCELL Technologies).

iPSC differentiation into macrophages
Macrophage differentiation from iPSCs was performed by first differentiating iPSCs into HSPCs using the STEMdiff Hematopoietic Differentiation kit (STEMCELL Technologies) according to the manufacturer's protocol, followed by culturing for 10-14 days in macrophage differentiation medium consisting of Iscove's Modified Dulbecco's Medium containing 10% fetal bovine serum (heat-inactivated embryonic stem cell qualified FBS; Atlanta Biologicals; R&D Systems), 1x antibiotic-antimycotic (Gibco; Thermo Fisher), and 100 ng/mL human macrophage colony-stimulating factor (M-CSF; PeproTech).

Intracellular Flow Cytometry
Cells were pelleted by centrifugation and incubated with Fixation and Permeabilization Solution (BD Biosciences) for 30 minutes on ice. The cells were then washed with 1X Permeabilization Wash Buffer (Biolegend) and stained with antibodies in FACS Buffer (1-2% FBS, 0.05% sodium azide, and 2-5 mM EDTA in PBS) at 4°C for 30-60 minutes. The stained cells were then washed with PBS or FACS buffer, pelleted, and resuspended at ~$1x10^6$ cells/ml in FACS Fix Buffer (FACS Buffer with or without 1% PFA) for flow cytometry analysis (Fortessa). The flow data was analyzed using FlowJo.

Western Blot
Cells were lysed with NuPage LDS sample buffer (Thermo Fisher Scientific) at the concentration of 10^5 cells per 15uL LDS supplemented with 10% BME and Benzonase Nuclease (Sigma Aldrich). iPSC macrophages were stimulated with IL-1β for 24 hours in cRPMI and then harvested for nuclear and cytoplasmic protein isolation (Abcam) according to the manufacturer's protocol. The samples were then denatured at 70°C. Protein lysates were separated by SDS-PAGE on 4-12% Bis-Tris pre-cast gels (Invitrogen) and transferred to a PVDF membrane (Invitrogen) by wet transfer. Membranes were then blocked in milk with 5% Tris-buffered saline with 0.01% Tween-20) TBST for an hour at room temperature and then incubated with primary antibody in milk or 5% BSA overnight at 4°C. The membrane was washed for 3 x 10 minutes with TBST at room temperature and then stained with HRP-linked secondary antibody in milk for 1 hour at room temperature. After 3 x 10 minute washes with TBST and 1 x 10 minute wash with PBS, the membrane was exposed to enhanced chemiluminescent (ECL) substrates (Thermo Fisher Scientific) and developed by film.

Cloning and Site-Directed Mutagenesis

The wild-type human IL37 gene (Genscript) was cloned into the pLV vector to produce pLV-WT-IL37 for subsequent transfection and transduction experiments using the In-Fusion HD Cloning Kit (Takara Clontech). IL37 with common variants was synthesized by IDT and also cloned into the pLV vector. Site-directed mutagenesis of T>C (p.I177T) was performed using the In-Fusion HD Cloning Kit (Takara Clontech) and following PCR primers (Sigma Aldrich): F: GGCGGCTCACCCCGGATGGTTCACCTGCAC and R: TTACAATTGCAGGAGGTGCAGGTGAACCAT. The PCR products were purified on a 1% agarose gel by gel electrophoresis and the desired mutagenized product band was cut out. The PCR product was purified using the NucleoSpin Gel and PCR Clean Up (Takara) from the InFusion Cloning Kit. The linearized, mutagenized product was ligated using the InFusion Enzyme (Takara) to generate the I177T mutant pLV-MUT-IL37 plasmid. Stellar cells (Takara) were transformed with the new plasmid by heat shock; the transformed cells were plated on ampicillin plates and incubated overnight at 37°C. Plasmid was extracted from individual colonies using the QIAprep Spin MiniPrep Kit (Qiagen). The mutation was confirmed by Sanger sequencing.

HEK293T Transfection and Jurkat and THP-1 Transduction

HEK293T cells were cultured in complete DMEM at 37°C in T75 flasks. $4x10^5$ cells in 2mL media were seeded into 6 well plates and grown overnight at 37°C. At 40-50% confluence, the cells were transfected with either pLV-CV-IL37, pLV-WT-IL37 or pLV-MUT-IL37 alone or with pSPAX2, and pMD2.g for lentivirus production. Transfections were performed in OPTI-MEM (Gibco) and GeneJuice Transfection Reagent (VWR) or Lipofectamine 3000 (ThermoFisher Scientific). Lentivirus was harvested at 24, 48, and 72 hours post-transfection. The viral supernatants were pooled and concentrated using Lenti-X concentrator (Clontech). Jurkat and THP-1 cells were separately cultured in complete RPMI. The cells were transduced with a ratio of 100:1 WT-IL37 or MUT-IL37 lentivirus: Jurkat cells or 10:1 lentivirus: THP-1 cells on retronectin-coated plates by spinfection. The cells were transduced for 72 hours in complete RPMI. Transduced cells were then selected for using puromycin selection. Supernatants of transfected and transduced cells were taken for IL-37 ELISA measurements.

Cycloheximide Chase Assay

HEK293T cells transfected with WT-IL37 or MUT-IL37 were cultured in complete DMEM at $1x10^6$ cells per mL and were treated with vehicle (complete DMEM) or cycloheximide (50 ug/mL, Sigma Aldrich) and MG132 (5 uM, Sigma Aldrich) for 10 or 20 hours. At each time point, the cells were harvested and subsequently analyzed by immunoblot.

IL-37 TNF Suppression Assay

THP-1 cells transduced with WT-IL37 or MUT-IL37 were cultured in complete RPMI at $3.5x10^5$ cells per mL. The cells were then differentiated with PMA (100 ng/mL, Sigma Aldrich) for 24 hours. Then PMA was removed by washing the adherent differentiated THP-1 cells. Next the cells were stimulated with a titrated dose of LPS for 4 hours in complete RPMI. The supernatants were then taken for ELISA measurements. iPSC-derived macrophages were cultured in complete IMDM and stimulated with LPS (100 ng/mL) for 4 hours, IL-1beta (10 ng/mL, PeproTech) for 24 hours, or IL-18 (10 ng/mL, PeproTech) for 24 hours. The supernatants were taken for ELISA or multiplex FACS bead-based cytokine measurements.

Enzyme-Linked Immunosorbent Assay (ELISA) and Multiplex Cytokine Assay

Supernatants were analyzed for IL-37 using IL-37 Human ELISA kit (Invitrogen) or Human IL-1F7 DuoSet ELISA kit (R&D Systems) and for TNF using Human TNF ELISA set (BD Biosciences). Supernatants were also analyzed for 13 different human inflammatory cytokines using a multiplexed flow cytometry bead-based approach (Biolegend).

SI Appendix References

11. A. M. Ellisdon et al., Homodimerization attenuates the anti-inflammatory activity of interleukin-37.
Sci. Immunol. 2, eaaj1548 (2017).
12. R. K. Merling et al. Transgene-free iPSCs generated from small volume peripheral blood non-mobilized CD34+ cells. Blood. 121(14): e98-e107 (2013).
